# What factors predict recurrence of a spontaneous pneumothorax?

**DOI:** 10.1186/1749-8090-7-112

**Published:** 2012-10-17

**Authors:** Hidetaka Uramoto, Hidehiko Shimokawa, Fumihiro Tanaka

**Affiliations:** 1 Second Department of Surgery, School of Medicine, University of Occupational and Environmental Health, 1-1 Iseigaoka, Yahatanishi-ku, Kitakyushu, 807-8555, Japan

**Keywords:** Spontaneous pneumothorax, Operation, VATS, Postoperative recurrence, Risk factor

## Abstract

**Background:**

The purpose of this retrospective study was to identify the risk factors for postoperative recurrence for the patients with a spontaneous pneumothorax (SP). A total of 214 patients were studied over a period of five years. Of these patients, 189 (88.3%) and 25 (11.7%) underwent video assisted thoracoscopic surgery (VATS) and an open approach for treatment, respectively. There were 35 (16.4%) postoperative recurrences.

**Methods:**

The data on patient characteristics, surgical details, and perioperative outcomes were analyzed. We compared the clinicopathological characteristics between recurrent and non-recurrent cases, and used logistic regression models to predict the risk factors for postoperative recurrence.

**Results:**

The differences in the age, gender, lesion site, location, ipsilateral SP (ISP), and contralateral SP (CSP) did not reach statistical significance between the two groups. However, the incidence of recurrence was higher in the subjects without any smoking history, and who had comorbidities, and a history of surgery for ISP. Concerning intraoperative factors, there were no significant differences with regard to the approach, buttress stapling, covering, surgeon, or length of the operation. The postoperative recurrence rate was higher in the patients who had been hand-stitched compared to those who had undergone instrument-based repair for blebs. There were no significant differences in the perioperative outcomes. The logistic regression models indicated that non smokers, those with comorbidities, and those who had previously undergone surgery for ISP had a higher rate of postoperative recurrence.

**Conclusions:**

We conclude that a history of no smoking, the existence of comorbidities, previous surgery for ISP, and hand stitching increase the risk of postoperative recurrence. Therefore, surgeons must be aware of these risk factors, and more carefully monitor such patients for recurrence.

## Background

Spontaneous pneumothorax (SP) remains a significant health problem because of the high recurrence rate during thoracic drainage and conservative treatments [1]. Even when surgical management is performed, the recurrence rate was estimated to be approximately 10%–20% [2]. Therefore, reinforcement of the visceral pleura around the staple line has been used in clinical practice [2]. Nevertheless, the best procedure has not yet been established, and the results are by no means satisfactory at present. The purpose of this study was to determine the risk factors for recurrent pneumothorax after surgery.

## Methods

### Patients and clinicopathological features

The institutional review board approved this study. From 2005 to 2010, 221 patients requiring surgery due to SP were included in this retrospective study conducted at the University of Occupational and Environmental Health. Preoperative investigations included chest radiographs and a high-resolution computed tomographic (CT) scan of the thorax. Six patients with ipsilateral lung cancer and 1 with lymphangioleiomyomatosis (LAM) were excluded from the analyses. Finally, a total of 214 patients were included in the present series. The indications for surgical treatment of patients with SP included a persistent air leak, tension pneumothorax, and obvious existence of bulla lesions at first presentation, hemopneumothorax, the occupation of patient, and ipsilateral or contralateral recurrences. Data were collected retrospectively for all patients and included a detailed history, age, sex, and smoking habits, past history of episodes of pneumothorax, treatment modalities, and surgical details. The postoperative variables assessed included the use of pleurodesis, postoperative complications, duration of chest drainage, and length of hospital stay. The patients were discharged from the hospital after surgery, and the follow-up data were collected via the outpatient department.

### Surgical procedures and management

All patients underwent general anesthesia and single lung ventilation with a double-lumen endotracheal tube and were placed in the lateral decubitus position. The 5- or 10-mm 0° video thoracoscope was inserted in the 7th intercostal space in the midaxillary line, making use of the hole made for the preoperative drainage tube. After inspection of the thoracic cavity, one or two additional ports were placed. The 5- or 10-mm working ports were placed in the 5th intercostal space between the scapula tip and anterior axillary line. The blebs were grasped with an endograsp and excised with the endo-GIA stapling device (Auto Suture Company Division, United States Surgical Corporation, Norwalk, USA) or ECHELON device (Ethicon Endo-Surgery, Inc; Cincinnati, Ohio) or were hand stitched. Buttress stapling was performed by polyglycolic acid (PGA) felt (Neoveil, Gunze Ltd, Kyoto, Japan) or DUET TRS (Covidien Autosuture, Mansfield, MA, 131 USA). The covering technique was performed using a PGA sheet (Neoveil) or surgical absorbable hemostat (Ethicon Endo-Surgery) with fibrin-glue (Beriplast P; Marburg, Germany or Bolheal: The Chemo-Sero-Therapeutic Research Institute, Kumamoto, Japan). An air leak test was performed under a pressure loading of 20 cm H_2_O. A chest tube was placed through one of the port sites. The surgery was performed by a surgical team including one surgical consultant, and procedures were selected based on the intraoperative situation. A total of 190 and 11 patients, repectively, underwent device and hand stitching for blebs. Both methods were applied for 9 cases, and 4 cases were treated by abrasion alone. Therefore, these 13 cases were excluded, and the surgical method was eliminated from our logistic regression models.

### Postoperative management and follow-up

All patients were extubated in the operating room. The chest tube was connected to an aspiration system, and negative suction of 5-or 10 cmH_2_O was applied. Intercostal drains were removed when the underlying lung was expanded with no residual air leak. Postoperative pain was primarily controlled by means of a thoracic epidural block, and oral analgesic medication was administered when necessary. Patients were discharged from the hospital when they were fully mobile. A follow-up was conducted for all patients. The mean follow-up and relapse free period were 391 and 67 days, respectively.

### Statistical analyses

Categorical variables were evaluated using the chi-square test and the *t*-test was utilized to analyze continuous variables between the two groups. The multivariate logistic regression analysis was used to evaluate independent associations. The odds ratio (OR) and 95% confidence interval (95% CI) were calculated for each variable. Differences were considered to be statistically significant for p-values < 0.05. The data were analyzed using the StatView software package (Abacus Concepts, Inc., Berkeley, CA).

## Results

### The relationships between recurrence and clinicopathological characteristics

In all, 214 patients were studied in this retrospective review. The clinicopathological characteristics of the patients are shown in Table 1. All of the patients were Japanese, consisting of 182 males and 32 females in this series, with a mean age of 38.8 years (range 14–94 years). A summary of the details of all of these cases is given in Table 1. The number of patients with and without a smoking habit was 75 and 139, respectively. One hundred three and 111 were right- and left-sided SP, respectively. One hundred seventy-seven (82.7%) were located on the top of the lung. Forty-four (20.6%) patients had a comorbid condition. The summary of comorbidities is given in Table 2. We compared the clinicopathological characteristics between recurrent and non- recurrent cases. One hundred thirty-four were first-episode patients, while 80 patients had either an ipsilateral or a contralateral recurrence. There were 35 (16.4%) postoperative recurrences. The differences in the age, gender, lesion site, location, ISP, and CSP, did not reach statistical significance between the two groups. However, the incidence of recurrence was higher in the subjects without a smoking history, those with comorbidities, and those who had previously undergone surgery for ISP.

**Table 1:** The relationship between recurence and clinicopathological characteristics

**Characteristic**	**Total**	**Rec**^**a**^	**%**	**Non-rec**	***p*-value**
All cases	214	35	16.4	179	
Age (years)					
<28	102	17	16.7	85	
≥28	112	18	16.1	94	0.906
Gender					
Male	182	28	15.4	154	
Female	32	7	21.9	25	0.360
Smoking					
Yes	75	7	9.3	68	
No	139	28	20.1	111	0.041
Lesion site					
Right	103	16	15.5	87	
Left	111	19	17.1	92	0.754
**Lesion location**					
Top	177	29	16.4	148	
**Other than top**	37	6	16.2	31	0.980
Comorbidities					
Yes	44	12	27.3	32	
No	170	23	13.5	147	0.028
ISP^b^					
Yes	50	9	18.0	41	
No	164	26	15.9	138	0.719
CSP^c^					
Yes	37	5	13.5	32	
No	177	30	16.9	147	0.607
Surgery for ISP					
Yes	22	8	36.4	14	
No	192	27	14.1	165	0.007

^a^ Rec: recurrence, ^b^ ISP: ipsilateral spontaneous pneumothorax, ^c^ CSP: contralateral spontaneous pneumothorax.

**Table 2:** The relationship between comorbidities and recurrence

**Comorbidity**	**Rec**	**Non-rec**
Interstitial pneumonia	7	9
HP^a^	0	3
BA^b^	0	3
Emphysema	0	3
Hematothorax	2	2
COPD^c^	0	2
Giant bulla	0	2
Hemophilia	0	2
Benign lung tumor	0	2
Empyema	2	0
RA^d^	3	0
Lung cancer	1	1
Catamenial pneumothorax	1	1
Extraocular myositis	0	1
Angiosarcoma	0	1
SLE^e^	0	1
AM^f^	0	1
Cystic disease of the lung	0	1

^a^ HP: hypersensitivity pneumonia, ^b^ BA: bronchial asthma, ^c^ COPD: chronic obstructive pulmonary disease, ^d^ RA: rheumatoid arthritis ^e^ SLE: systemic lupus erythematosis, ^f^AM: atypical mycobacteriosis, Some subjects had several comorbidities.

### Surgical data and perioperative results

One hundred eighty-nine (88.3%) subjects underwent VATS, while an open thoracotomy was carried out on 25 patients. Concerning the intraoperative factors, there were no significant differences in the approach, buttress stapling, covering, surgeon, or length of the operation. The postoperative recurrence rate was higher in subjects who underwent hand stitching than machine stitching for blebs (Table 3). There were no significant differences in the perioperative outcomes (Table 4). All complications are listed in Table 5. The postoperative course was uneventful in 199 (93.0%) patients and 15 (7.0%) complications were observed, while no intraoperative or in-hospital deaths were observed.

**Table 3:** The relationship between recurence and intraoperative factors

**Characteristic**	**Total**	**Rec**	**%**	**Non-rec**	***p*-value**
Approach					
VATS	189	31	16.4	158	
Thoracotomy	25	4	16.0	21	0.959
Methods					
Device	190	29	15.3	161	
Hand stitch	11	5	45.5	6	0.009
Buttress stapling					
Yes	27	5	18.5	22	
No	187	30	16.0	157	0.745
Covering					
Yes	173	30	17.3	143	
No	41	5	12.2	36	0.423
Surgeon					
Consultant	103	19	18.4	95	
Trainee	111	16	14.4	84	0.426
Length of operation (min)		120.7 (40–270 )		126.0 (35–315)	0.630

**Table 4:** Periopetative outcomes

**Characteristic**	**Total**	**Rec**	**%**	**Non-rec**	***p*-value**
Pleurodesis					
Yes	15	4	26.7	11	
No	199	31	15.6	168	0.263
Postoperative complication					
Yes	15	4	26.7	11	
No	199	31	15.6	168	0.263
Drainage period (days)		1.914 (1–4)		2.257 (1–9)	0.073
Hospital stay (days)		13.3 (2–140)		8.0 (2–140)	0.128

**Table 5:** Morbidty/mortality

**Complication**	**Rec**	**Non-rec**
Uneventful	31	168
Air leak	4	11*
Empyema	0	1*
Mortality	0	0
In-hospital death	0	0

*: One subject had a postoperative complication of both an air leak and empyema.

### The risk factors for recurrent SP

Logistic regression models indicated that a history of non-smoking, the existence of comorbidities, and a previous operation for ISP were predictive factors for postoperative recurrence (Tables 6 and 7).

**Table 6:** A univariate analysis of the factors contributing to the recurrence

**Variables**	**OR**^**a**^	**95% CI**^**b**^	**p-value**
Age: ≤28	1.044	0.506-2.156	0.906
Gender: male	0.649	0.256-1.646	0.363
Smoking: no	2.450	1.015-5.917	0.046
Lesion: top	1.012	0.387-2.646	0.980
Comorbidities: yes	2.398	1.0815.319	0.031
ISP: yes	1.165	0.506-2.681	0.719
CSP: yes	0.765	0.276-2.128	0.608
Operation for ISP: yes	3.497	1.339-9.091	0.011
Approach: VATS	1.029	0.331-3.205	0.959
Buttress stapling: no	0.841	0.295-2.392	0.745
Covering: no	0.662	0.240-1.828	0.426
Surgeon: trainee	0.745	0.360-1.540	0.427
Pleurodesis: yes	1.972	0.590-6.579	0.271
Complication: yes	2.652	0.466-15.152	0.271

^a^ OR: Odds ratio, ^b^ 95% CI: 95% confidence interval.

**Table 7:** The multivariate analysis of the factors contributing to the recurrence

**Variables**	**OR**	**95% CI**	**p-value**
Age: ≤28	1.277	0.489-3.333	0.617
Gender: male	1.237	0.366-4.183	0.732
Smoking: no	2.717	1.017-7.257	0.046
Lesion: top	1.531	0.484-4.844	0.469
Comorbidities: yes	3.413	1.236-9.433	0.018
ISP: yes	0.862	0.3615-2.364	0.773
CSP: yes	0.428	0.129-1.420	0.166
Operation for ISP: yes	4.695	1.486-14.925	0.008
Approach: VATS	1.802	0.464-6.999	0.395
Buttress stapling: no	0.724	0.232-2.262	0.578
Covering: no	0.594	0.193-1.825	0.363
Surgeon: trainee	0.787	0.332-1.867	0.587
Pleurodesis: yes	1.420	0.238-8.475	0.700
Complication: yes	1.763	0.133-23.255	0.667

## Discussion

The present study clearly demonstrated four major findings. First, a non-smoking history was an independent factor predicting postoperative recurrence. This finding might seem to be contrary to logic, because cigarette smoking increases the risk for SP [3]. The trapping of distal air because of smoking-induced bronchiolar inflammation, thus leading to alveolar overdistension and rupture, may also be a contributing factor [3]. In fact, smoking cessation has been associated with a decreased risk of recurrent SP over a follow-up period of several years [4]. However, in our series, a current smoking habit was unexpectedly associated with a favorable result with regard to recurrence compared to a lack of smoking. This discrepancy may be due to (1): the formation of new blebs due to overdistension after surgery for non-smokers (2): the existence of an entirely-different mechanism for the development of bullous lesions between natural and postoperative SP and (3) other unknown mechanisms. Bense et al. reported that subpleural blebs or bullae, which are designated as emphysema-like changes, are seen in 75–100% of patients with SP, even in non-smoker [5]. These findings were consistent with our results.

Second, the postoperative recurrence rate was higher in patients with comorbidities than in those without. This finding seems to be reasonable because there are different mechanisms underlying he development of bullous lesions between primary and secondary SP [1]. Interestingly, there might be an indirect association between SP and systemic diseases such as collage disease with regard to direct lung complications, such as interstitial pneumonia or chronic obstructive pulmonary disease (COPD).

Third, a history of previous surgery for ISP was associated with a higher rate of recurrence. New bullous lesions may have arisen due to postoperative overexpansion of the residual lung parenchyma due to volume loss from the pulmonary resection [6]. Unexpectedly, CSP did not affect the rate of postoperative recurrence, although Huang et al. previously reported that contralateral bleb formation was significantly associated with contralateral recurrence of SP [7]. As a result, a complicated relationship might therefore exist between unilateral/ bilateral SP and the pre/postoperative state.

Finally, the postoperative recurrence rate was higher in the patients who underwent hand stitching for blebs than those who underwent device-driven repair. These result suggested that the remaining bullous lesion might be at high risk because of previous failures of the non-resection methods, such as ligation and looping, which are known to be related to a high recurrence rate [6,8]. No statistically significant differences in recurrence were seen in any other intraoperative factors. These findings might suggest that since thoracoscopy has recently become more popular, and most thoracic surgeons are already familiar with the procedure, it should therefore be used whenever possible [9].

There are several limitations that must be taken into account when considering the present findings; including the retrospective nature of the study and the fact that it was carried out at a single institution. Further, university hospitals have a tendency to collect cases with high comorbidities. To overcome these limitations, prospective studies in a larger cohort of patients is necessary to clarify the risk factors for recurrence and to define the optimal surgical technique to suppress the recurrence of SP.

## Conclusion

In conclusion, the current results revealed several clinical factors that may be useful markers for predicting postoperative recurrence in patients with SP following surgery. Surgeons should therefore exercise additional caution in patients with these risk factors, and a more rigorous follow-up should also be considered for such patients.

## Abbreviations

SP: Spontaneous pneumothorax; VATS: Video-assisted thoracoscopic surgery; ISP: Ipsilateral spontaneous pneumothorax; CSP: Contralateral spontaneous pneumothorax; CT: Computed tomography; LAM: Lymphangioleiomyomatosis; PGA: Polyglycolic acid; OR: Odds ratio; CI: Confidence interval; COPD: Chronic obstructive pulmonary disease.

## Competing interests

Dr. Uramoto, Shimokawa, and Tanaka have no competing interests and conflict of interst or financial ties to disclose.

## Authors’ contributions

This report reflects the opinion of the authors and does not represent the official position of any institution or sponsor. The contributions of each of the authors were as follows: HU were responsible for reviewing previous research, journal hand searching, and drafting report. HS was responsible for reviewing previous research and journal hand searching. FT was responsible for project coordination. All authors have read and approved the final manuscript.

## References

[B1] Tschopp JM Rami-Porta R Noppen M Astoul P Management of spontaneous pneumothorax: state of the art Eur Respir J 2006 28 637 650 10.1183/09031936.06.00014206 16946095

[B2] Muramatsu T Nishii T Takeshita S Ishimoto S Morooka H Shiono M Preventing recurrence of spontaneous pneumothorax after thoracoscopic surgery: a review of recent results Surg Today 2010 40 696 699 10.1007/s00595-009-4208-1 20676850

[B3] Schramel FM Postmus PE Vanderschueren RG Current aspects of spontaneous pneumothorax Eur Respir J 1997 10 1372 1379 10.1183/09031936.97.10061372 9192946

[B4] Ayed AK Bazerbashi S Ben-Nakhi M Chandrasekran C Sukumar M Al-Rowayeh A Risk factors of spontaneous pneumothorax in Kuwait Med Princ Pract 2006 15 338 342 10.1159/000094266 16888390

[B5] Bense L Lewander R Eklund G Hedenstierna G Wiman LG Nonsmoking, non-alpha 1-antitrypsin deficiency-induced emphysema in nonsmokers with healed spontaneous pneumothorax, identified by computed tomography of the lungs Chest 1993 103 433 438 10.1378/chest.103.2.433 8432133

[B6] Inderbitzi RG Leiser A Furrer M Althaus U Three years’experience in video-assisted thoracic surgery (VATS) for spontaneous pneumothorax J Thorac Cardiovasc Surg 1994 107 1410 1415 8196381

[B7] Huang TW Lee SC Cheng YL Tzao C Hsu HH Chang H Contralateral recurrence of primary spontaneous pneumothorax Chest 2007 132 1146 1150 10.1378/chest.06-2772 17550937

[B8] Takeno Y Thoracoscopic treatment of spontaneous pneumothorax Ann Thorac Surg 1993 56 688 690 10.1016/0003-4975(93)90953-F 8379772

[B9] Oka S Uramoto H Hanagiri T Successful extirpation of thoracic pleural lipoma by single port thoracoscopic surgery Asian J Surg 2011 34 140 142 10.1016/j.asjsur.2011.08.006 22208690

